# Occupational Exposure among Electronic Repair Workers in Ghana

**DOI:** 10.3390/ijerph19148477

**Published:** 2022-07-11

**Authors:** Stine Eriksen Hammer, Stephen L. Dorn, Emmanuel Dartey, Balázs Berlinger, Yngvar Thomassen, Dag G. Ellingsen

**Affiliations:** 1 National Institute of Occupational Health, Gydas vei 8, 0363 Oslo, Norway; berlinger.balazs@univet.hu (B.B.); yngvar.thomassen@stami.no (Y.T.); dag.ellingsen@stami.no (D.G.E.); 2 Team Analytics and Environment, Münster Electrochemical Energy Technology, University of Münster, Schlossplatz 3, D-48159 Münster, Germany; stephen.dorn@uni-muenster.de; 3 Department of Chemistry Education, Akenten Appiah-Menka University of Skills Training and Entrepreneurial Development, Mampong P.O. Box 40, Ghana; emmldartey@yahoo.co.uk

**Keywords:** exposure assessment, electronic repair workers, remanufacturing, soldering, metals, rare earth elements

## Abstract

Electronic repair workers may be exposed to lead, mercury, cadmium and other elements including rare earth elements used in electronic equipment. In this study, repair work took place in small repair shops where, e.g., televisions, radios, video players, compact discs and computers were repaired. Personal full-shift air samples of particulate matter were collected among 64 electronic repair workers in Kumasi (Ghana) and analysed for 29 elements by inductively coupled plasma mass spectrometry. Results showed that air concentrations of all elements were low. The highest air concentration was measured for iron with a geometric mean concentration and geometric standard deviation of 6.3 ± 0.001 µg/m^3^. The corresponding concentration of Pb and Hg were 157 ± 3 ng/m^3^ and 0.2 ± 2.7 ng/m^3^, respectively. The cerium concentration of 5 ± 2 ng/m^3^ was the highest among the rare earth elements. Source apportionment with ranked principal component analysis indicated that 63% of the variance could be explained by the repair and soldering of electronic components such as batteries, magnets, displays and printed circuit boards. An association between concentrations of lead in the workroom air and lead in whole blood was found (Pearson’s correlation coefficient r = 0.42, *p* < 0.001). There was, however, no statistically significant difference between whole blood lead concentrations in the workers and references indicating that lead did not exclusively originate from occupational exposure.

## 1. Introduction

Electronic equipment may contain a number of elements such as cadmium (Cd), lead (Pb) and mercury (Hg), as well as some rare earth elements (REEs) [1]. Presently, there is much regulatory focus on recycling, remanufacturing and disposal of electronic waste [2]. In Ghana, about 215,000 tons of electronics were imported in 2009, of which 70% was second-hand and 14% went directly to be repaired [3]. Repair work is regarded as a remanufacturing process, where broken parts are manually disassembled and replaced with functional components. Remanufacturing electronic equipment is important in order to reduce the environmental burden from the disposal of hazardous materials [4], but also to reduce the need for elements that are not easily available, such as REEs [5]. 

In remanufacturing work, occupational exposure may take place during, e.g., sorting, shredding, desoldering and dismantling, as well as reassembling and soldering. In informal recycling, materials are also melted or burnt [6]. Exposure during electronic remanufacturing may occur by inhalation, dermal contact, and ingestion. Studies of occupational exposure to elements during general repair work of electronic equipment are scarce [7,8,9]. There has, however, been more focus on formal and informal recycling [10]. A study of workers from the large electronic waste dumping site at Agbogbloshie (Ghana) revealed that workers who self-reported sorting electronic waste had around twice as high Pb whole-blood concentrations (B-Pb) compared to workers who did not [11]. Burning electronic waste has been associated with the highest B-Pb concentrations in informal recycling studies [11,12]. Higher concentrations of Hg in urine, indium (In) in plasma and urine and B-Pb have been reported in formal recycling workers compared to office workers [13]. In an exposure study on workers in sheltered workshops in Germany, no difference was found in the exposure to aluminium (Al), antimony (Sb), arsenic (As), beryllium (Be), Cd, chromium (Cr), cobalt (Co), nickel (Ni) and Hg compared to a reference group of office workers [14]. The exposure among the Swedish recycling workers [13] was 5–300 times higher than the German sheltered workshop workers. 

Rare earth elements have been widely used in electronics since yttrium and europium (Eu) were applied commercially in colour televisions in the 1960s to obtain the red colour (Y_2_O_2_S:Eu^3+^) [15]. In electronic devices, REEs can be found in e.g., batteries, magnets, printed circuit boards (PCBs), lamps, cathode-ray tubes and liquid-crystal displays [16]. The use of REEs, especially neodymium (Nd), praseodymium (Pr) and dysprosium (Dy), is expected to increase due to the demand for green energy products such as magnets for hybrid and electric cars, as well as wind turbines in addition to general electronic equipment [5]. The presence of REEs in different electronics provides the opportunity to use these elements as source indicators, e.g., lanthanum (La) and Nd were found in mobile displays from different manufacturers while Pr and Dy were used in old Nokia phones [1]. Scandium (Sc) and cerium (Ce) were detected in most PCBs [17]. Europium, terbium (Tb) and yttrium (Y) are often present in lamps used in electronic equipment [18,19]. 

Occupational exposure to metals such as Pb and Hg has been much studied in contrast to REEs [20]. In general, toxicological studies of REEs have shown low toxicity [21]. However, few cases of pulmonary fibrosis and pneumoconiosis have been reported among workers in different occupations [22,23,24,25]. 

Occupational exposure among Pb battery and electronic repair workers in Kumasi (Ghana) was assessed with biomonitoring by Dartey et al. The electronic repair workers restored electronic equipment such as TVs, radios, video players and computers. Their work included dismantling, soldering, welding and reassembling the equipment. The electronic repair workers had higher concentrations of, e.g., Cd in blood (B-Cd) and Sn in urine (U-Sn) than the reference group. Dartey et al. [8] suggested that this was related to soldering during repair work. The aim of this study is to investigate the concentration of relevant elements including some REEs in full-shift air filters collected among the same electronic repair workers and to compare this to biomonitored metals in the same population. Source apportionment was performed to identify possible sources of airborne particulate matter (PM). The study adds to the knowledge on the concentrations of several elements one may be exposed to, as well as the possible sources of exposure during electronic repair work.

## 2. Materials and Methods

### 2.1. Site

The study was carried out in Bantama located right in the central hub of Kumasi, which is the second most populated city in Ghana, with more than 2 million inhabitants. Kumasi covers an area of 254 km^2^ [26]. The electronic repair workshops were about 12 m^2^ and employed an average of 3–5 workers. The buildings had only natural ventilation, either semi-open buildings or closed buildings with only window ventilation. The main job tasks of the electronic repair workers were dismantling, soldering and welding electronic equipment such as television sets, radio sets, video cassette recorders, computers and compact disks. Most workers were found to eat and drink at the workplace. No proper hand washing facility nor clothes changing facility were observed in the workshops. Personal protective equipment at the workplace was not used.

### 2.2. Design

Inclusion criteria for participating in the study were age between 18 and 50 years old, and at least one year of employment as an electronic repair worker. In total, 85 male electronic repair workers in 21 different electronic workshops were invited and 64 of them volunteered to participate. 

### 2.3. Sample Collection

Air and biological sampling were performed on consecutive days in 2011 among 64 electronic repair workers. Aerosol sampling cassettes (Millipore, Bedford, MA, USA) equipped with 25 mm polyvinyl chloride membrane filters (5 µm pore size) were used to collect PM in work room air by personal sampling during a 6 h full-shift period with the use of Sidekick air pumps (SKC Ltd., Dorset, UK). The pumps were calibrated to an air flow rate of 2 L/min using a rotameter (Vögtlin instruments, Basel, Switzerland). Field blank filter samples were also included. Whole blood was collected by trained health staff from the cubital vein the same day as the participants brought a first voided morning urine sample to the examination. All 64 participants volunteered to give a blood sample, but only 59 participants gave a urine sample. The biological samples were collected, prepared (blood samples were conserved with ultrapure nitric acid and urine was heated) and analysed by inductively coupled plasma sector-field mass spectrometry as described in Dartey et al. [8]. 

### 2.4. Air Filter Dissolution and Measurement of Elements

Air filters were transferred to 15 mL polypropylene tubes (Sarstedt, Nümbrecht, Germany) and 1 mL of *aqua regia* was added containing a 3:1 mixture (vol.) of puriss p.a. ≥ 37% HCl and ≥65% HNO_3_ (Sigma-Aldrich, St. Louis, MO, USA). An internal standard solution containing 4 µg/mL of rhenium (Re), tellurium (Te) (Spectrapure Standards AS, Oslo, Norway) and ^74^Se (99.9% of ^74^Se) (STB Isotope Germany GmbH, Hamburg, Germany) was added to all samples prior to heating. After heating to 90 °C for 60 min in a laboratory oven, the samples were diluted to 10 mL with deionized water (Milli Q System, 18 MΩ cm, Millipore Corp., Billerica, MA, USA).

The air filter solutions were analysed with an Agilent 8800 triple quadrupole inductively coupled plasma mass spectrometer (ICP-MS, Agilent Technologies, Santa Clara, CA, USA) operating in the oxygen reaction mode. Acid matrix-matched multielement calibration standards were prepared by dilution of certified primary standards (Spectrapure Standards AS, Oslo, Norway). The following isotopes were measured (oxygen reaction mass shift is marked with **r**, on-mass was measured otherwise): ^75**r**^As, ^209^bismuth (Bi), ^114^Cd, ^140**r**^Ce, ^59^Co, ^63^copper (Cu), ^52^Cr, ^153**r**^Eu, ^158**r**^Gd, ^69^gallium (Ga), ^202^Hg, ^56^iron (Fe), ^139**r**^La, ^55**r**^manganese (Mn), ^144**r**^Nd, ^60^Ni, ^206+207+208^Pb, ^195^platinum (Pt), ^141**r**^Pr, ^121^Sb, ^45**r**^Sc, ^107^silver (Ag), ^120^Sn, ^159**r**^Tb, ^203^thallium (Tl), ^51**r**^vanadium (V), ^182^tungsten(W), ^89**r**^Y and ^66^zinc (Zn). The element concentrations of Pb were calculated using the sum of the isotopic net signals (marked with +). A correction factor was applied for ^114^Cd (−0.0269cps ^118^Sn) to eliminate the ^114^Sn isobaric overlap. ^74^Se was the selected internal standard for elements measured in oxygen mass-shift mode including ^60^Ni. ^187^Re was selected for oxygen mass-shift mode for elements heavier than ^60^Ni. ^130^Te was selected as an internal standard for elements measured on-mass. Quality assurance of the measurements was carried out using surface water quality control material at two different concentration ranges (SPS-SW1 and SPS-SW2, Batch 124 and Batch 137, Spectrapure Standards AS, Oslo, Norway). In the quality control samples, most elements were within 5% of the target value. However, Co and Cu were within 10% and Fe was within 20% for SPS-SW1 and As, Mn and V were within 25% of the target values of the surface water quality control materials. The limit of detection (LOD) in ng/m^3^ was calculated as three times the standard deviation of eight field blank filters using an average air volume of 0.72 m^3^.

### 2.5. Statistics

Shapiro–Wilk’s normality test showed that all variables were significantly different from the normal distribution. Thus, geometric means (GM) and standard deviations (GSD) are presented. The median, GM, GSD, minimum and maximum concentrations are presented for all elements with less than 66% censored data. Elements with more than 66% censored data (Cr, Eu, Gd, Pr, Pt, Tb and W) were removed from the statistical analysis. Values below LOD were substituted with ½ LOD for Bi (13%), Cd (36%), Co (3%), Cu (9%), Hg (30%), La (3%), Nd (11%), Ni (55%), Sc (41%), Tl (17%) and Y (25%).

Rank principal component analysis (PCA) was conducted on 22 elements with varimax rotation in the CRAN package ‘psych’ [27]. An initial analysis was run to obtain eigenvalues for each component in the data, and Kaiser’s criterion was applied to select the number of components. Ranked data were used for PCA to avoid the problems of substitution, or exclusion of variables with a high fraction > 30% up to 66% of censored data [28]. 

**Figure 1:**
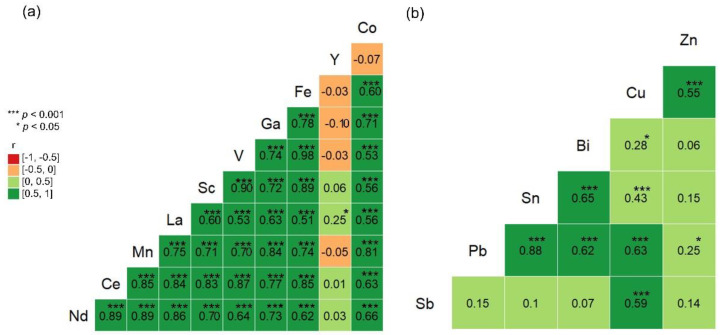
Pearson’s correlation coefficients calculated between air concentrations of all metals grouped in rotated component (RC)1 (**a**) and RC2 (**b**).

**Figure 2:**
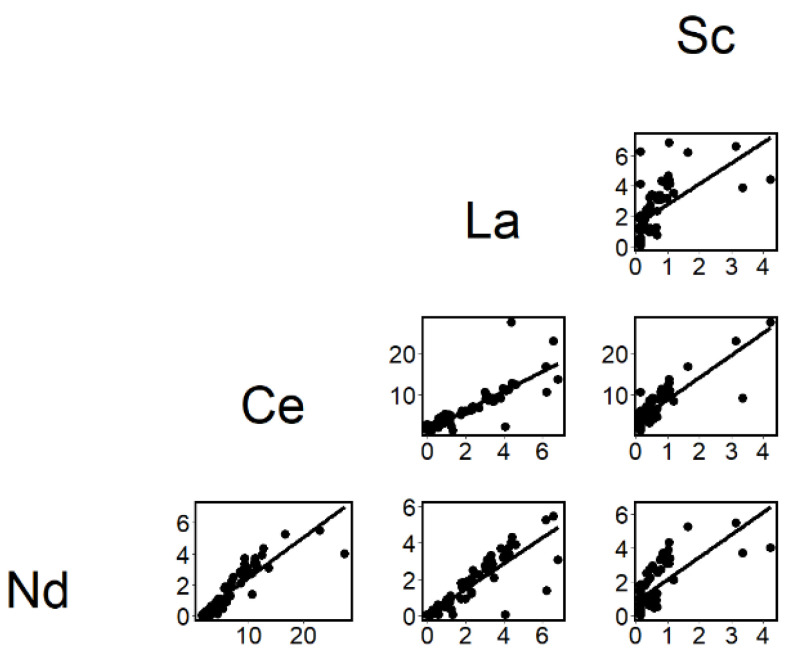
The associations between the concentrations of the rare earth elements Sc, La, Ce and Nd in rotated component 1 (ng/m^3^).

**Figure 3:**
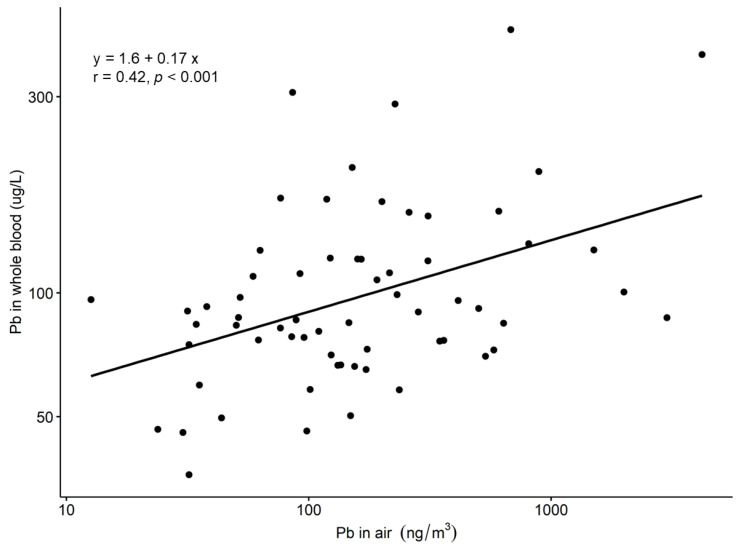
The association between the concentrations of Air-Pb and B-Pb (*n* = 64). The regression equation and Pearson’s correlation coefficient are shown in the figure.

Associations (Figure 1, Figure 2 and Figure 3) were assessed with least square regression analysis. Pearson’s correlation coefficient was calculated as the measure of the association. A *p*-value < 0.05 was considered statistically significant. 

All statistical calculations were performed in R studio [29] version 3.5.3. The figures were made with the additional CRAN packages in R studio: ‘ggplot2’ [30], ‘GGally’ [31] and ‘ggpubr’ [32]. 

## 3. Results

The full-shift air concentrations of elements are shown in Table 1. The concentrations of the measured elements in the workroom air are low, but concentration ranges are substantial. Iron, followed in decreasing order by Zn, Pb, Sn, Mn and Cu, dominated the elemental composition of the PM. The most abundant of the REEs is Ce, followed by La, Nd, Y and Sc. 

Four RCs exceeded eigenvalues of 1 according to the Kaiser’s criterion which in combination explained 79% of the variance. The RCs from rank PCA and the first four RCs with possible sources of the included elements are presented in Table 2. The cut point for an element within the component was set to 0.7. The elements in the first RC may be related to repair of batteries and magnets as well as work on steel. The elements in the second RC are hypothesised sources from repair work on PCBs such as soldering. 

The correlation matrices for RC1 and RC2 show the Pearson’s correlation of the different coefficients calculated between air concentrations of elements (Figure 1). Most elements within RC1 correlate significantly with each other except for Y. Yttrium is together with Eu and Tb widely used in lamps. However, Eu and Tb had more than 66% censored data and could therefore not be included in the statistical analysis.

The associations between the REEs element in RC1, which had a Pearson’s r > 0.5, are shown in Figure 2. The correlations between La, Ce and Nd fit the regression lines well. However, Sc had more values below LOD. Still, positive significant correlations were observed between Sc, La, Ce and Nd, respectively, when removing the values below LOD.

The associations between the air concentrations of Pb and Cd and the respective concentrations in whole blood, as well as the air concentrations of Sn and Pb and the respective concentrations in urine, were investigated. A slight positive association (Pearson’s r = 0.41, *p* < 0.001) between the concentrations of B-Pb and Air-Pb was observed (Figure 3). No significant association was found between Sn and Cd and the respective concentrations in urine or whole blood.

## 4. Discussion

### 4.1. Workroom Air Concentrations

The full-shift air concentrations of all elements were low, and well below occupational regulatory limits (OSHA) and recommended exposure limits (NIOSH) [33]. This shows that electronic repair work such as soldering, welding or grinding in the studied workshops contaminated the workroom air only with trace amounts of PM-containing elements during a workday. More than 50% relative abundance of the elemental concentrations measured was Fe. Typically, Fe is the main constituent of many components used in electronic equipment, e.g., steel and magnets. Cadmium, Cu, Hg and Pb air concentrations are between a factor of 100–12,500 times higher in formal recycling than among the electronic repair workers in this study [13]. It should be noted, however, that air concentrations of Hg may have been underestimated since volatile Hg species were not collected with the sampling method used. Iron, Zn and Pb were present with the highest relative abundance in the electronic recycling dust [13] similar to our results. The air concentrations of As, Cd, Co, Ni and Sb were 10–50 times higher in sheltered workshops in Germany [14] compared to this study. The GM and GSD of the Air-Pb concentrations (0.157 ± 0.003 µg/m^3^) are similar to the Air-Pb concentrations measured in a laboratory chamber study during intensive soldering on microelectronics (0.18 ± 0.08 µg/m^3^) [34]. Based on statistical modelling, they concluded that this air concentration would not result in increased B-Pb concentrations. 

PM in ambient air may contribute to the workroom air concentrations. To our knowledge, there is no scientific literature addressing element concentrations in ambient air in Kumasi. Therefore, concentrations measured in the electronic workshops were compared to ambient air concentrations in other parts of the world. The measured workroom air concentrations of Cd, Co, Cu, Fe, Mn, Pb and Zn were a factor of about 5–10 times higher than levels measured in studies of ambient air in North Carolina (US), Turin (Italy), Daejeon (Korea) and Krakow (Poland) [35,36,37,38]. Compared to PM_2.5_ at two sites (campus area and downtown) in Beijing (China), air concentrations of Fe and Cu were higher by a factor of 2 and 6 in the electronic repair shops, but Mn and Ni were similar [39]. However, the Air-Pb concentrations were around 50% of the air concentrations measured at those. Concentrations of REEs among the electronic repair workers were between a factor of 5–10 times higher compared to the amounts in PM in a heavily urbanised and industrialised area in The Netherlands [40]. Thus, the concentrations of several elements measured in this study indicate occupational activities as the main source of exposure. 

### 4.2. Ranked Principal Component Analysis

Rank PCA resulted in four RCs explaining 79% of the variance. The first RC explained 38% of the variance and included Nd, Ce, Mn, La, V, Sc, Ga, Fe, Co and Y. Elements typical of steel such as Fe, Mn, Co and V were found in this RC which could indicate the origin of electronic equipment. All these elements typically present in steel correlated significantly with each other (Figure 1). This RC also included battery components such as Mn, Ce, La, Nd and Co, but did not include Ni which is the main component in Ni metal batteries (NiMH) [41]. The negligible amount of Ni in the samples may explain why this element was not included in RC1. Lanthanum and Nd are often used to produce colours in smartphone displays [1], but the main elements in such aluminosilicate glass, silicon and aluminium, were not analysed in the present study. Iron and Nd are magnet components used in Nd-Fe-Boron magnets. Magnet components are used in, e.g., hard discs, optical drives, smartphones and loudspeakers [42]. Lanthanum and Ce were associated with NiMH alloys, metallurgical sources, automobile catalysts, polishing powders, and glass additives in a study of the end use of REEs worldwide [43]. In summary, this RC may include elements related to welding on steel and alloys, replacement of batteries and/or dismantling and repair of, e.g., displays and magnet components in computers and phones.

The second RC contained elements typically used in soldering such as Cu, Sn and Pb [44], as well as elements used in PCBs such as Sb and Zn [1,45,46,47]. Antimony is present in organic epoxy resins used on PCBs in computers. Soldering in electronic repair work is performed, e.g., to change components on PCBs, connect PCBs together and/or to repair wires. A similar principal component, including Cd, chlorine, Co, Cu, In, Pb, Rh, rubidium, Sb, Sn, sulphur and Zn, was found in soil samples collected inside and in the vicinity of the large electronic waste dumping site at Agbogbloshie [48]. The authors concluded that soil from the burn site at Agbogbloshie was enriched with chemical elements from Sn-Pb solder and Pb-free solder.

The two last RC included only one element each, Cd in RC3 and Ni in RC4. Additionally, they both include a high load of censored data, 36% (Cd) and 55% (Ni), respectively. Source apportionment from one element with a relatively high load of censored data is speculative. Both elements are found in batteries; Cd is often used in soldering material and coatings. 

### 4.3. Associations between Workroom Air and Biological Concentrations

Occupational exposure routes to metals such as Pb, Sn and Cd may include inhalation, transfer from hand to mouth with subsequent gastrointestinal exposure and skin absorption [49,50,51,52]. Hand hygiene among workers handling electronic equipment may be a source of the enhanced B-Pb, B-Cd and U-Sn, as 81% of the electronic repair workers and 78% of the control group reported that they did not wash their hands with soap before eating [8]. The association between Air-Cd and -Sn with B-Cd and U-Sn was not statistically significant. However, we found an association between the Air-Pb and B-Pb (Figure 2). A similar association was observed among female electronic solderers by Mohammadyan et al. [53]. However, they were exposed to considerably higher mean Air-Pb (0.09 ± 0.01 mg/m^3^) concentration than the electronic repair workers. Exposure to soldering fumes is dependent on several factors such as the composition of the solder, temperature and ventilation. Another study measured very low concentrations of Air-Pb even during intensive soldering [34]. In a further study of soldering, the exposure to Air-Pb was significantly higher for solderers than the control group, mean of 0.57 µg/m^3^ and 0.0067 µg/m^3^, respectively [54]. However, in that study, Air-Pb did not correlate with B-Pb which could have been influenced by individual exhaust fans used to remove the fumes, or that the production rate was lower than normal on the day of air sampling, as suggested by the authors. Electronic workers, who were soldering, had slightly higher concentrations of B-Pb than unexposed referents (6.1 µg/dL versus 4.6 µg/dL of Pb) [55]. These studies imply that exposure to Pb during soft soldering is marginal due to the low temperatures applied. However, another main hazard exists by the decomposition of the soldering flux which is commonly rosin-based. Such fume is a potent respiratory sensitiser and has been shown to cause occupation asthma, but exposures are seldom measured [56,57].

Dartey et al. [8] did not observe a significant difference in the B-Pb concentrations between electronic repair workers and the reference group. That reference group comprised workers from two different areas of Kumasi. However, when the electronic repair workers are only compared to the referents recruited from the same area of the electronic repair workshops, then the electronic repair workers have slightly higher B-Pb concentrations (GM B-Pb 97 µg/L, range 36–473 µg/L, and GM 85 µg/L, range 34–276 µg/L, *p*-value = 0.20). 

Generally, the B-Pb concentrations in the studied populations were relatively high with similar levels measured in Western countries when alkylated Pb compounds were used as antiknocking agents in gasoline [58,59,60]. However, the use of leaded gasoline was banned in Ghana years before this study was conducted and phased out between 2002 and 2004 [61]. Hackman et al. [62] have discussed the possibility of illegal use of leaded fuel, based on measurements of exhaust from cars in the Accra region in Ghana. Biomonitoring of Pb in gas-station workers in the Accra region revealed a non-significantly (*p* = 0.061) higher mean B-Pb concentration (3.4 µg/dL) than the control group (3.1 µg/dL) [63]. Still, these concentrations are substantially lower than the GM B-Pb of 102 µg/L concentrations in the electronic repair workers and referents [8]. Elevated concentrations of Pb in groundwater were observed in the Oti community area, Kumasi, Ghana [64]. This was suggested as a consequence of dumping lead batteries, pipes and paints at the Oti landfill site. Additionally, Pb concentrations have been measured above the threshold limit value of surface soil at an industrial site in Kumasi, Ghana [65]. However, the Pb concentration was below the threshold limit value around the residential sites. More research is needed to reveal the causes of the high B-Pb levels in the studied population.

## 5. Conclusions

This study shows that air concentrations of the measured elements were low. Still, rank PCA analysis resulted in components which may indicate work operations or product groups that can be related to various types of repair work. The first RC included elements related to repair work on steel, batteries, and magnets. The second RC included elements which may indicate soldering or work on PCBs. The third and fourth RC included only one element with a relatively high load of censored data which makes these components more difficult to interpret. Biological monitoring showed that the Air-Pb concentrations correlated with B-Pb concentrations. Still, the elevated amounts of B-Pb cannot be explained solely by occupational exposure as the reference group had only slightly lower B-Pb concentrations [8].

## Figures and Tables

**Table 1:** Full-shift geometric mean (GM) air concentrations [ng/m^3^] and geometric standard deviation (GSD) of elements collected among electronic repair workers, (*n* = 64).

Element	Median	GM ± GSD	Minimum	Maximum	LOD ^a^	% < LOD
Ag	1.0	1 ± 3	0.2	76	0.04	0
As	1.9	2 ± 2	0.3	14	0.2	0
Bi	0.4	0.3 ± 4	<LOD	29	0.05	13
Cd	0.3	0.4 ± 3	<LOD	13.4	0.02	36
Ce	5.4	5 ± 2	1.4	28	0.07	0
Co	1.4	1 ± 2	<LOD	4.8	0.2	3
Cu	78	81 ± 2	<LOD	583	31	9
Fe ^b^	6.5	6.3 ± 0.001	1.9	41	0.3	0
Ga	3.1	3 ± 2	0.8	8.2	0.08	0
Hg ^c^	0.2	0.2 ± 3	<LOD	1.4	0.1	30
La	1.8	1 ± 3	<LOD	6.8	0.05	3
Mn	94	90 ± 2	23	269	3.7	0
Nd	1.1	1 ± 5	<LOD	5.5	0.04	11
Ni	<LOD	27 ± 2	<LOD	272	27	55
Pb	147	157 ± 3	13	4.2 × 10^3^	0.9	0
Sb	8.0	9 ± 3	1.0	764	0.1	0
Sc	0.4	0.4 ± 2	<LOD	4.2	0.3	41
Sn	107	116 ± 4	2.4	4.3 × 10^3^	1.3	0
Tl	0.06	0.05 ± 2	<LOD	0.4	0.03	17
V	13	13 ± 2	3	92	0.3	0
Y	0.9	0.5 ± 7	<LOD	23	0.05	25
Zn	271	340 ± 2	67	4.3 × 10^3^	23	0

^a^ Limit of detection, ^b^ µg/m^3^, ^c^ non-volatile.

**Table 2:** Rank principal component analysis with varimax rotation.

Rotated Component	Proportion Variance	Elements	Possible Work Sources
RC1	0.38	Nd, Ce, Mn, La, Sc, V, Ga, Fe, Y, Co	Batteries, magnets, and steel
RC2	0.25	Sb, Pb, Sn, Bi, Cu, Zn	Soldering, printed circuit boards
RC3	0.06	Cd	
RC4	0.10	Ni	

## Data Availability

The dataset is available upon request to Stine Eriksen Hammer (stine.hammer@stami.no).
